# Interval Training Improves Depressive Symptoms But Not Anxious Symptoms in Healthy Women

**DOI:** 10.3389/fpsyt.2019.00661

**Published:** 2019-09-12

**Authors:** Ricardo Borges Viana, Paulo Gentil, João Pedro Araújo Naves, Ana Cristina Silva Rebelo, Douglas Assis Teles Santos, Marco Aurélio Oliveira Braga, Claudio Andre Barbosa de Lira

**Affiliations:** ^1^Faculty of Physical Education and Dance, Federal University of Goiás, Goiânia, Brazil; ^2^Institute of Biological Sciences, Federal University of Goiás, Goiânia, Brazil; ^3^College of Physical Education, State Bahia University, Teixeira de Freitas, Brazil

**Keywords:** exercise, depression, anxiety, mood disorders, mental health

## Abstract

**Background:** Despite important advances in the relationship between exercise and mood disorders, especially regarding moderate-intensity continuous training, there is a lack of information about the chronic effects of interval training protocols. We compared the effects of two different interval training protocols [sprint interval training (SIT) and high-intensity interval training (HIIT)] on depressive and anxious symptoms in healthy women.

**Methods:** Thirty-six women were randomly allocated to HIIT (n = 18) or SIT (n = 18) groups and performed 24 training sessions over 8 weeks (thrice a week). Levels of state–trait anxiety and depressive symptoms were evaluated using State–Trait Anxiety Inventory and Beck Depression Inventory, respectively, before and after training intervention.

**Results:** Two-way analysis of variance (ANOVA) did not reveal a significant effect of time (p > 0.05), group intervention (p > 0.05), or time × group interaction (p > 0.05) on state–trait anxiety; however, two-way ANOVA showed a significant effect of time on depressive symptoms (p = 0.025) but not group effect (p = 0.548) or time × group interaction (p = 0.373). Depressive symptoms of the participants in both HIIT and SIT groups were reduced from baseline, (Δ_HIIT_) −17.5 ± 27.9% and (Δ_SIT_) −28.6 ± 47.5%, respectively.

**Conclusion:** HIIT and SIT groups similarly improved depressive symptoms but not anxiety levels in healthy and physically active young adult women.

## Introduction

Physical exercise is recognized as a non-pharmacological tool for prevention and treatment of noncommunicable diseases such as diabetes mellitus, hypertension, and mood disorders (1, 2). With regard to the latter, a large amount of literature shows that physical exercise improves mood disorders, mainly depression (1) and anxiety (2). Briefly, these studies showed that physical exercise is effective in improving levels of depression (1) and anxiety (2) symptoms in clinical and nonclinical populations. Furthermore, physical exercise as a non-pharmacological treatment has been shown to be superior to control interventions and sometimes comparable to medication for mild and moderate depression (2, 3).

Overall, the main advantage of exercise as a therapeutic tool for mood disorders is that physical exercise is a low-cost intervention that does not present side effects like pharmacological treatment and that can be offered to everyone, since it does not present contraindications (4). This is particularly important given the high cost associated with cognitive behavior treatment and poor access of patients to trained cognitive behavior health professionals (4). Despite the indisputable positive effect of exercise on health status and mood disorders, the number of people that are sedentary is high (5), and the main reason used to explain sedentary behavior by persons is the lack of time and/or pleasure (6).

For this reason, sport and exercise researchers are devoted to investigating alternatives to increase the effectiveness and efficiency of physical exercise (4). In this context, interval training appears as an alternative way (low volume of exercise) to induce similar or greater performance and cardiovascular adaptations than traditional moderate-intensity continuous exercise, which makes it more time-efficient (7); thus interval training can be a useful tool to increase adherence of a population to a regular physical exercise program (6). Briefly, studies showed that interval training can cause physiological adaptations, such as increase in maximal oxygen uptake $\left(\dot{V}{\text{O}}_{2}\max\right)$ (8) and changes in markers of adiposity (9) and body composition (10).

Interval training is defined, according to Gibala et al. (11), as “alternating periods of relatively intense exercise with periods of lower-intensity effort or complete rest for recovery.” The two most-used forms of interval training are the sprint interval training (SIT) and high-intensity interval training (HIIT) (11). SIT refers to protocols that are performed at “supramaximal” or “all-out” efforts at intensities corresponding to loads/intensities greater than those associated to a maximum oxygen uptake $\left(i\dot{V}{\text{O}}_{2}\max\right)$ obtained in an incremental test (12). On the other hand, HIIT is typically described as a training stimulus with target intensity between 80% and 100% of maximal heart rate (HRmax) (12).

Despite important advances in the relationship between exercise and mood disorders, especially regarding moderate-intensity continuous training (13), there is, so far as we are aware, a lack of information about the long-term effects of different interval training protocols. The few studies that investigated the acute and long-term effects of interval exercise on psychological outcomes focused on the perceived exertion, affect, and arousal (14, 15). Previous studies reported that depending how HIIT protocols are designed, HIIT might result in high levels of enjoyment (16–20), high adherence (17), and low perceived exertion (16) when compared to traditional exercise protocols (continuous training protocols). For instance, Jung et al. (18) showed that interval training elicited greater enjoyment than continuous training protocols and that half of the participants reported a preference to engage in interval training. One year later, Jung et al. (17) showed that prediabetic individuals had a greater adherence to HIIT [4–10 × 1 min at ∼90% of peak heart rate (HRpeak) interspersed by 1 min at low intensity] than to moderate-intensity continuous training (20–50 min at ∼65% HRpeak) after 10 sessions of exercise performed over a 12-day period.

As there is a high prevalence of depression and anxiety and it is increasing, especially in women (21), novel treatments that simultaneously improve depressive and anxious symptoms and physical health status are needed (22). Furthermore, about 33.7% of the population was or will be affected by one sort of anxiety disorder during their lifetime (21), and this prevalence is more evident in teenagers, young adults (23), and women, who are 60% more likely than men to experience anxiety over their lifetime (24). Therefore, our study aimed to compare the effects of 8 weeks of two different interval training protocols (HIIT and SIT) on depressive and anxious symptoms in healthy women. Since physical exercise can increase neurotrophin levels (25), and HIIT has been shown to improve depression and neural plasticity in the hippocampus in poststroke depression rats (26), we hypothesized that HIIT and SIT would improve the depressive and anxious symptoms in healthy women. In addition, due the fact that both HIIT and SIT are performed at high intensity, we hypothesized that no difference will be found in the depressive and anxious symptoms between HIIT and SIT over intervention.

## Methods

### Participants

**Figure 1:**
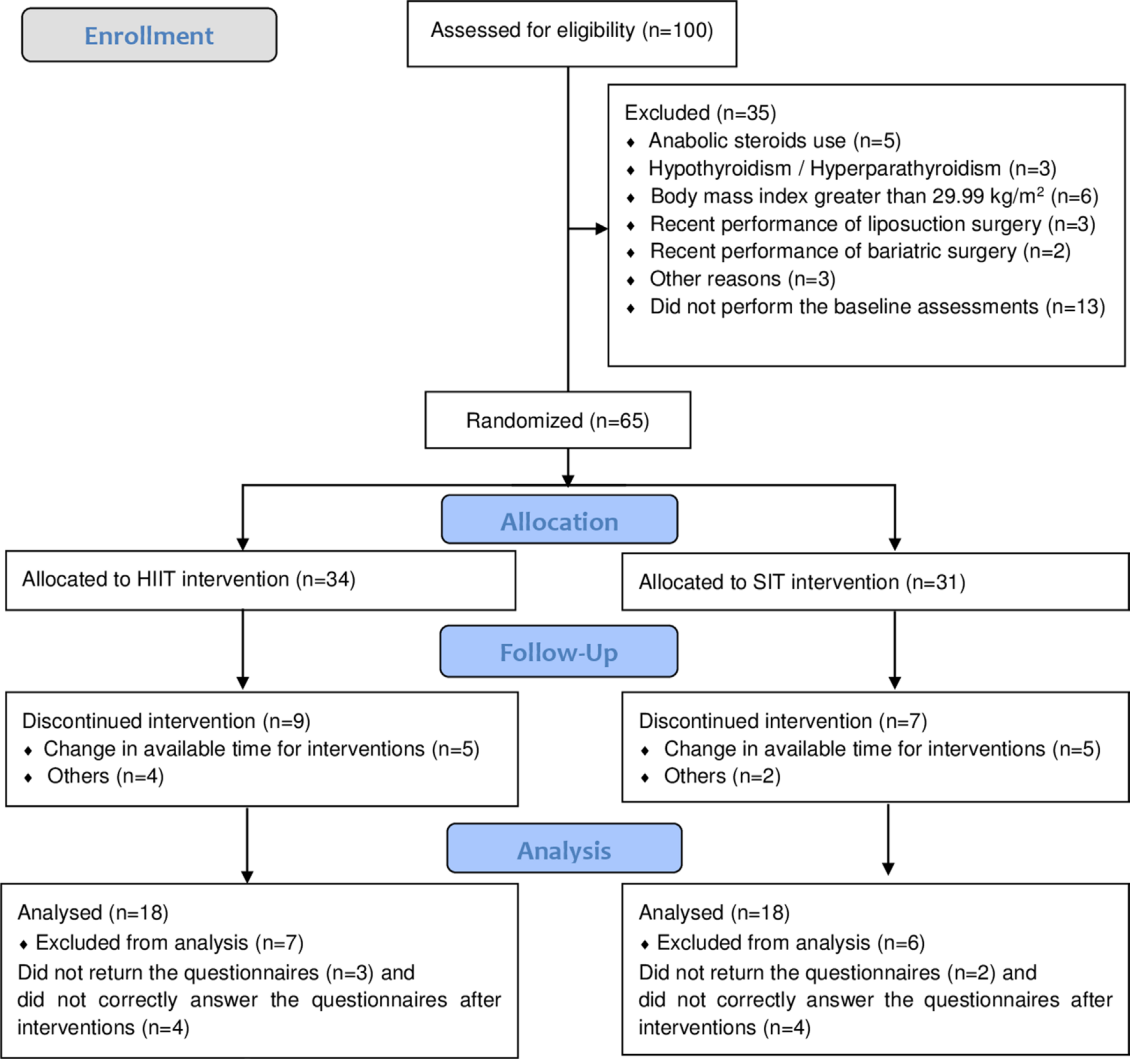
Diagram flow of participants. HIIT, high-intensity interval training; SIT, sprint interval training.

Participants of the present study were also involved in another study, aimed to compare the long-term effects of SIT and HIIT on body composition and cardiorespiratory fitness (9). Initially, 100 women were recruited. Participants were recruited through social media and word of mouth. To be included in the present study, the participants needed to: i) be women; ii) have a body mass index greater than 18.5 and less than 30.0 kg/m^2^; iii) be physically active (equal or greater than 150 min/week); iv) be pre-menopause; v) not use any thermogenic and/or stimulants; and vi) have no HIIT and SIT experience during the last 6 months. Exclusion criteria were: i) contraindications for performing physical activity evaluated by the Physical Activity Readiness Questionnaire (PAR-Q) (27) and ii) any intervention for body mass loss. After the application of the inclusion and the exclusion criteria, 35 participants were excluded. Of the 65 remaining participants, 34 and 31 were allocated randomly to the HIIT and SIT groups, respectively. Sixteen participants withdrew from the study, and 13 did not return the questionnaires or did not correctly answer the questionnaires after interventions. Therefore, 36 healthy and physically active women were included in the analysis (HIIT, n = 18, 30.9 ± 6.9 years, 1.65 ± 0.05 m, 63.5 ± 7.9 kg, and 23.4 ± 2.5 km/m^2^; and SIT, n = 18, 29.2 ± 6.3 years, 1.65 ± 0.05 m, 67.5 ± 8.7 kg, and 24.9 ± 3.6 kg/m^2^). All reasons for participants’ exclusion and dropout from interventions are presented in the **Figure 1**.

All participants provided signed informed consent, in compliance with the Declaration of Helsinki (28). All experimental procedures were approved by the University Research Ethics Committee (nº 1.542.353).

### Study Design

This was a longitudinal study organized in four consecutive phases. Firstly, the participants were submitted to an anamnesis (PAR-Q) 2 weeks before the beginning of the training period. Secondly, in the week before the training period, all the participants were submitted to a graded exercise test to determine peak oxygen uptake $\left(\dot{V}{\text{O}}_{2}\text{peak}\right)$ and HRpeak, and self-answered the State–Trait Anxiety Inventory (STAI) and Beck Depression Inventory (BDI) to assess anxious and depressive symptoms, respectively. Thirdly, the participants performed 24 sessions spread over 8 weeks (three times *per* week: Mondays, Wednesdays, and Fridays), with recovery intervals of 48–72 h. The participants performed a variation of the HIIT protocol proposed by Wisløff et al. (29) or the SIT protocol proposed by Gibala et al. (30) on a mechanical braked cycle ergometer (Evolution SR, Schwinn, USA) and were asked to not perform any other type of exercise apart from the physical exercise proposed by the study. When three or fewer non-consecutive training sessions were missed by a participant, the training sessions were replaced at the end of the period. However, in case four or more sessions were missed, the participant would be excluded from the study. It is noteworthy that no participant missed four or more sessions. Finally, in the week after the last training session, the participants performed all post-intervention evaluations.

### Cardiorespiratory Graded Exertion Test

In order to ensure that interval training protocols were effective in promoting the expected training adaptations, we submitted the participants to a graded exercise test on an electromagnetic braked cycle ergometer (CG04, Inbramed, Brazil) to determine their $\dot{V}{\text{O}}_{2}\text{peak}$ and HRpeak. Briefly, following a 2 min warm-up (50 W at 80 rpm), the resistance was increased by 25 W every 1 min until volitional exhaustion or the point at which the participant was not able to maintain a pedal cadence of at least 50 rpm. After exhaustion, the participants performed a recovery for 2 min at 50 W and a pedal cadence of 50 rpm. A metabolic system (VO2000, MedGraphics, USA) was used to $\dot{V}{\text{O}}_{2}\text{peak}$ measure, and heart rate (HR) was constantly monitored by an HR monitor (Polar RS800, Kempele, Finland). The Borg Scale (6 to 20) (31) was used to evaluate the rating of perceived exertion at each stage of the test.

### Interval Training Protocols

As described by Naves et al. (9), SIT training sessions consisted of a warm-up of 5 min (load and cadence self-selected), following by four repeated 30 s all-out cycling efforts, alternated with 4 min of passive recovery or light cycling with no load (30). The workload was adjusted to each participant to maintain frequency ≥60 rpm. HIIT training sessions consisted of a warm-up of 5 min at 50% of HRpeak, followed by four repeated 4 min efforts at 90% to 95% of HRpeak, alternated with 3 min of recovery at 50% to 60% of HRpeak (29). The workload was kept constant during the efforts, being adjusted (more or less) only when the HR moved outside the established zone. The cadence was kept free during the recovery and the load decreased to the minimum. Each participant was directly supervised by one investigator (experienced with interval training prescription) during all training sessions, and standardized verbal stimuli were provided. SIT and HIIT sessions lasted 23 and 33 min, respectively.

### Anxious and Depressive Symptoms

STAI (32) validated for the Brazilian population (33) was used to evaluate anxious symptoms. Scores lower than 30, of 31 to 49, and of 50 to 80 indicate the presence of low, intermediate, and high level of anxiety, respectively (34). The STAI was used since it is the most-cited anxiety instrument in the context of physical exercise and sport science (35), translated to 30 languages (34) (including Portuguese language), and due to its easy application.

BDI (36) validated for the Brazilian population (37) was used to evaluate depressive symptoms. Scores from 0 to 9, 10 to 16, 17 to 29, and 30 to 63 indicate minimal, mild, moderate, and severe depressive symptoms (38). The BDI was used since it is one of the most commonly used screening instruments for depression worldwide, translated to 18 languages (including Portuguese language), and due to its easy application (39).

### Statistical Analysis

Data are presented as mean, standard deviation, and absolute and relative frequencies. Data normality was tested by Shapiro–Wilk test. A two-way analysis of variance (ANOVA) [group intervention (HIIT × SIT) × time (pre × post)] was used for comparing group means on variables. As, $\dot{V}{\text{O}}_{2}\text{peak}$ state–trait anxiety, and depressive symptoms changes were non-normally distributed, we used Spearman’s rank correlation (coefficient reported as r). Statistical Package for the Social Sciences (version 21.0, IBM Corp., Armonk, NY, USA) was used to perform all statistical analysis. We set our significance level α at 0.05. A blinded technician performed all analyses.

## Results

### Training Adherence

Only two participants (one from the SIT group and other from the HIIT group) missed one training session and needed to replace this session at the end of the training period.

### State–Trait Anxiety

Two-way ANOVA did not reveal a significant effect of time [F (1, 34) = 0.043; p = 0.837], group [F (1, 34) = 0.103; p = 0.749], or time × group interaction [F (1, 34) = 0.279; p = 0.599] on state anxiety. Two-way ANOVA also did not reveal a significant effect of time [F (1, 34) = 0.690; p = 0.409], group [F (1, 34) = 1.269; p = 0.264], or time × group interaction [F (1, 34) = 0.124; p = 0.725] on trait anxiety (**Table 1**). At baseline, 77.8% (HIIT, n = 13; SIT, n = 15) and 75% (HIIT, n = 14; SIT, n = 13) of participants presented a moderate level of state and trait anxiety, respectively. After the intervention, these proportions changed to 72.2% (HIIT, n = 14; SIT, n = 13) and 63.9% (HIIT, n = 13; SIT, n = 10), respectively.

**Table 1:** Cardiorespiratory fitness, anxiety levels, and depressive symptoms of the participants before and after HIIT and SIT intervention.

Variables	HIIT		SIT		p		
	Pre	Post	Pre	Post	Group effect	Time effect	Group × time interaction
$\dot{V}{\text{O}}_{2}\text{peak}$ (ml.kg^−1^.min^−1^)	38.6 ± 7.6	43.7 ± 5.4	33.8 ± 6.5	37.3 ± 6.7	0.001	0.008	0.603
$i\dot{V}{\text{O}}_{2}peak$ (watts)	163 ± 30	168 ± 27	140 ± 23	150 ± 21	0.001	0.207	0.729
State–trait anxiety							
State anxiety	39.1 ± 10.3	39.8 ± 11.0	38.6 ± 8.6	38.8 ± 11.1	0.749	0.837	0.909
Trait anxiety	39.4 ± 7.6	38.2 ± 10.7	43.0 ± 11.0	40.1 ± 11.6	0.264	0.409	0.725
BDI score	12.6 ± 5.1	10.5 ± 6.4	14.9 ± 7.2	10.1 ± 6.9	0.548	0.025	0.373

Data are expressed as means ± standard deviation. HIIT, high-intensity interval training; SIT, sprint interval training; $\dot{V}{\text{O}}_{2}\text{peak}$, peak oxygen uptake; $i\dot{V}{\text{O}}_{2}\text{peak}$ (watts), intensity associated at peak oxygen uptake; BDI, Beck Depression Inventory; p, values are from two-way analysis of variance (ANOVA) (2 × 2).

### Depression

Two-way ANOVA revealed a significant effect of time on depressive symptoms [F (1, 34) = 5.238; p = 0.025] but not group effect [F (1, 34) = 0.365; p = 0.548] or time × group interaction [F (1, 34) = 0.805; p = 0.373] (**Table 1**). Depressive symptoms of the participants in both HIIT and SIT groups were reduced from baseline, (Δ_HIIT_) −17.5 ± 27.9% and (Δ_SIT_) −28.6 ± 47.5%, respectively. At baseline, 30.5% of participants (HIIT, n = 5; SIT, n = 6) presented a normal level of depression; however, more than half of participants (61.1%, n = 22) presented a normal level of depression after HIIT and SIT interventions.

### Cardiorespiratory Fitness

Two-way ANOVA showed a significant effect of time [F (1, 34) = 7.554; p = 0.008] and group [F (1, 34) = 12.846; p = 0.001] on $\dot{V}{\text{O}}_{2}\max$ but not time × group interaction [F (1, 34) = 0.274; p = 0.603]. $\dot{V}{\text{O}}_{2}\max$ of the participants in both HIIT and SIT groups increased from baseline [(Δ_HIIT_) 16.3 ± 25.7% and (Δ_SIT_) 12.0 ± 17.6%, respectively] (**Table 1**). No significant correlation was found between changes in $\dot{V}{\text{O}}_{2}\text{peak}$ and changes in depressive symptoms (r = −0.17, p = 0.335), changes in trait anxiety levels (r = −0.22, p = 0.200), or changes in state anxiety levels (r = −0.02, p = 0.927).

## Discussion

Our study aimed to compare the effects of 8 weeks of two different interval training protocols (HIIT and SIT) on depressive and anxious symptoms in healthy women. Our findings partially confirm the initial hypothesis, since both groups similarly improved depressive symptoms but not anxious symptoms of the participants. We also found that cardiorespiratory fitness similarly improved with both protocols.

We found that $\dot{V}{\text{O}}_{2}\text{peak}$ of the participants in both HIIT and SIT groups increased without a significant difference between then. Previously, some studies showed that interval training increases $\dot{V}{\text{O}}_{2}\max$. Trilk et al. (40) found an increase in $\dot{V}{\text{O}}_{2}\max$ of 12% in sedentary and overweight/obese women submitted to an SIT protocol [4 to 7 sprints of 30 s (cycling against a load corresponding to 5% of body mass) interspaced by 4 min of active recovery (low cadence at 0% body mass)] three times *per* week for 4 weeks. Duffield et al. (41) found an increase in $\dot{V}{\text{O}}_{2}\max$ of ∼21% in physically active females after 8 weeks (three times *per* week) of HIIT protocol (four increased progressively to 12 intervals of 2 min cycling at 130–180% of power at lactate threshold interspaced by 1 min of passive recovery). Therefore, our data are in accordance with the literature.

Regarding depressive and anxious symptoms, seminal data show that exercise, over the short or long term, is an effective tool to improve depression and anxiety (42). In the current study, we found that both interval protocols adopted were effective in improving depressive symptoms but not anxious symptoms. The impact of exercise in anxiety disorders is usually smaller than that observed for depressive symptoms (42), which can partially explain our findings (significant improvements in depressive symptoms but not in anxiety levels). In addition, dose–response between amount of exercise and reductions in anxiety is not well established (2). Another possible explanation for the absence of changes in anxiety levels is that we assessed non-clinical participants. Indeed, at baseline, 88.9% and 88.9% of participants from the HIIT group presented, respectively, state and trait anxiety of low or middle levels, and 94.4% and 77.8% of participants from the SIT group presented, respectively, state and trait anxiety of low and middle levels. Therefore, the absence of changes in anxiety levels is expected due to “ground effect.” Additionally, studies testing the long-term effects of different interval training protocols on depressive and anxious symptoms of patients with anxiety disorders are needed.

Specifically regarding depressive symptoms, notwithstanding that we have evaluated non-clinical participants, we found that both interval protocols led to significantly improved BDI scores. Unlike what was found for anxious symptoms, for depressive symptoms, more than 60% of participants presented some degree of depression at baseline. After the training protocol, about 60% of participants were classified as normal in BDI score. Therefore, interval training protocols applied in the current study were effective in managing depressive symptoms. So far as we are aware, less is known about the long-term effects of interval training protocols in a non-clinical population; thus, our results are a novelty.

As reported by Schuch et al. (43), when considering the available data, aerobic exercise performed three sessions *per* week over 12 to 24 weeks, delivered in groups, and supervised by an instructor seems to have greater efficacy. In spite of the elevated physiological strain associated with interval training (15, 44) and higher contribution from anaerobic metabolism for energy production, interval training is predominantly aerobic (45). Also, training protocols were delivered in groups by a specialized instructor.

Previous studies explain this positive outcome of exercise in depression levels due to physiological and neurobiological mechanisms (46). Briefly, Heinzel et al. (46) reported that aerobic training improves cardiorespiratory fitness, which stimulates neurotrophins (47, 48), stress-associated hormone cortisol (49), the oxygen and energy supply of the brain (50, 51), and the synthesis and release of endocannabinoids (central and peripheral). These changes might inhibit the hypothalamic–pituitary–adrenal axis, leading to a reduction in cortisol release (52) and, consequently, of the psychological stress response (53, 54), and positive affect (55). As recent studies in major depressive disorders (25, 56–58) showed improvements in neurotrophin levels through physical exercise, this mechanism may be related to physical exercise interventions. As reported earlier, Luo et al. (26) found that interval training (in specific HIIT) improved depression and neural plasticity in the hippocampus in poststroke depression rats. Moreover, TaheriChadorneshin et al. (59) found that SIT provided greater increases in brain-derived neurotrophic factor in the brains of rats after 6 weeks than intensive endurance training. In the current study, $\dot{V}{\text{O}}_{2}\text{peak}$ increased in both interval training groups; however, we did not find any significant correlation between the $\dot{V}{\text{O}}_{2}\text{peak}$ changes and the changes in the other variables investigated. Therefore, experimental studies evaluating the possible mechanisms related to depressive symptom reductions and interval training are needed.

Although we did not assess enjoyment levels, there is evidence that interval training resulted in high enjoyment (14, 60), high adherence (17), and low ratings of perceived exertion (60) in novice exercisers when training involved shorter-duration interval bouts (equal to or less than 1 min) performed at intensities equal or closer to $i\dot{V}{\text{O}}_{2}\max$. Likely, the alternation of stimuli and the shorter total time of the session make interval training (e.g., HIIT and SIT) less monotonous. Thus, it is possible that the greater enjoyment associated with HIIT and SIT may be relevant for improving exercise adherence (an important barrier associated with binomials depression and exercise). Thus, interval exercise must be considered by health professionals as an effective way of treatment.

Finally, some researchers advocate that exercise prescription for depression should be affect-based (61). Moreover, according to Murri et al. (22), “this method expands the traditional focus of exercise prescriptions from the dual goal of maximizing fitness gains while minimizing risk to a model that also aims to ensure that participants consistently derive pleasant affective experiences.”

### Limitations of the Study

Our study is not without limitations. Firstly, we did not measure enjoyment after training sessions and total intervention. Secondly, the participants were a non-clinical sample. Although we did not recruit a clinical sample, we believe that any small improvement in depressive and anxious symptoms in a non-clinical population might be an indication that the interventions applied may also provide improvements in a clinical sample. Furthermore, although the lack of reductions in the anxiety scores suggested a possible ground effect, the presence of this effect might suggest that the anxiety-reducing effect of HIIT and SIT might only be noticeable in those with a high level of distress. Thirdly, we did not evaluate the behavioral activation. Fourthly, we did not evaluate psychological and/or physiological mechanisms. Finally, there was no control of the menstrual period during the measurements of anxiety levels and depression symptoms. Therefore, future studies are needed to examine these putative underlying mechanisms.

## Conclusions

Eight weeks of HIIT and SIT similarly improved depressive symptoms but not anxiety levels in young adult women. In addition, both interval training protocols improved cardiorespiratory fitness. Therefore, as interval training protocols are performed at high intensities, studies investigating the physiological and psychological mechanisms related to intense exercise and depression constitute a fundamental step towards the broad use of interval training protocols (e.g., HIIT and/or SIT) as an alternative non-pharmacological treatment of depression in the field.

## Data Availability

The datasets generated for this study are available on request to the corresponding author.

## Ethics Statement

The studies involving human participants were reviewed and approved by Comitê de Ética em Pesquisa da Universidade Federal de Goiás (protocol number: 1.542.353). The patients/participants provided their written informed consent to participate in this study.

## Author Contributions

RV, PG, and CL designed the study. JN and MB collected the data. RV analyzed the data. RV and CL wrote the manuscript. RV, PG, AR, DS, and CL revised the manuscript. All authors read and approved the final manuscript.

## Funding

This study was funded by Fundação de Amparo à Pesquisa do Estado de Goiás-FAPEG/Brazil (grant number 201210267001056) and by Conselho Nacional de Desenvolvimento Científico e Tecnológico-CNPq/Brazil (grant numbers 475774/2012-4 and 304435/2018-0).

## Conflict of Interest Statement

The authors declare that the research was conducted in the absence of any commercial or financial relationships that could be construed as a potential conflict of interest.
